# Efficacy and tolerability of α-galactosidase in treating gas-related symptoms in children: a randomized, double-blind, placebo controlled trial

**DOI:** 10.1186/1471-230X-13-142

**Published:** 2013-09-24

**Authors:** Giovanni Di Nardo, Salvatore Oliva, Federica Ferrari, Saverio Mallardo, Giovanni Barbara, Cesare Cremon, Marina Aloi, Salvatore Cucchiara

**Affiliations:** 1 Pediatric Gastroenterology and Liver Unit, Sapienza – University of Rome, Rome, Italy; 2 Department of Medical Science, University of Bologna, Bologna, Italy

**Keywords:** Gas-related symptoms, Bloating, α-galactosidase, Children

## Abstract

**Background:**

Gas-related symptoms represent very common complaints in children. The reduction of gas production can be considered as a valuable target in controlling symptoms. α-galactosidase has been shown to reduce gas production and related symptoms in adults. To evaluate the efficacy and tolerability of α-galactosidase in the treatment of gas-related symptoms in pediatric patients.

**Methods:**

Single center, randomized, double-blind, placebo-controlled, parallel group study performed in tertiary care setting. Fifty-two pediatric patients (32 female, age range 4–17) with chronic or recurrent gas-related symptoms were randomized to receive placebo (n = 25) or α-galactosidase (n = 27). Both treatments were given as drops or tablets, according to body weight for 2 weeks. The primary endpoint was the reduction in global distress measured by the Faces Pain Scale-Revised (FPS-R) at the end of treatment compared to baseline. Secondary endpoints were the reduction in severity and frequency of gas-related symptoms as recorded by parents and/or children.

**Results:**

α-galactosidase significantly reduced global distress (*p* = 0.02) compared to placebo. The digestive enzyme decreased the number of days with moderate to severe bloating (p = 0.03) and the proportion of patients with flatulence (*p* = 0.02). No significant differences were found for abdominal spasms and abdominal distension. No adverse events were reported during treatment.

**Conclusions:**

Although larger and longer trials are needed to confirm this result, α-galactosidase seems to be a safe, well tolerated and effective treatment for gas-related symptoms in the pediatric population.

**Trial registration:**

ClinicalTrials.gov, NCT01595932

## Background

Most patients with functional gastrointestinal disorders, including those with irritable bowel syndrome, complain of gas-related symptoms, e.g. bloating, abdominal distention, and flatulence [1]. Indeed, more than 20% of the general population suffers from abdominal pain or discomfort and 16% from bloating or distension [2] with no evidence of any inflammatory, anatomic, metabolic or neoplastic process that explains the subject’s symptoms. Intestinal gas-related symptoms are generally not considered to be a severe medical problem but can negatively affect quality of life, even in pediatric age [3].

The mechanism underlying gas-related disorders is not well understood. As a result, the best treatment is still not well defined [4,5]. Bloating is the sensation of trapped gas and tightness in the abdomen which is exacerbated by meals and fluctuates in intensity during the day. It may be associated with distension (an objective increase in waist size) and flatulence. Flatulence or gas build up in the intestine is typically caused by the fermentation of undigested food and may cause voluntary or involuntary passing of gas [6].

Therefore, agents that decrease the amount of gas in the intestine may represent an effective therapeutic strategy for the management of gas-related symptoms when their cause depends, at least in part, on gas hyper-production.

There are different strategies that may reduce intestinal gas. Current approaches are based on dietary changes and life-style factors; administration of simethicone; activated charcoal and probiotics [7-12]. However in most cases results are unsatisfactory.

The food enzyme, *α*-galactosidase, derived from *Aspergillus niger* mold, acts in the gastrointestinal tract by breaking down specific non-absorbable oligosaccharides before they are metabolized by colonic bacteria [13]. As a result, this agent may be useful to reduce the amount of fermentable substrates in the colon and prevent the overproduction of gas associated with meals. Obviously the same effect could be obtained by selecting foods which do not contain those specific non-absorbable oligosaccharides. However this selection is not easy and may lead to the elimination of healthy food (cereals, legumes and vegetables).

There are some data supporting the efficacy of α-galactosidase in reducing breath hydrogen (a measure of intestinal gas production) and in preventing gas formation in the colon and related disturbances [14,15]. However, the studies are available only in adult populations.

The aim of this study was to evaluate the efficacy and tolerability of α-galactosidase in pediatric patients with predominant gas-related symptoms.

## Methods

This was a single center, randomized, double blind, placebo-controlled, parallel group study performed in tertiary care setting. Fifty two pediatric out patients (32 female) with gas-related disturbances at least once per week over the last 12 weeks, were included in the study.

Exclusion criteria were: 1) any suspected episodes of hypersensitivity/allergy; 2) any chronic organic disorders, as assessed by full clinical history and examination, and supported by normal results of initial limited laboratory investigation including complete blood cell count, erythrocyte sedimentation rate, C-reactive protein, blood glucose, amylase and lipase, tissue transglutaminase antibodies with total serum IgA; 3) inability of the parent to comprehend the full nature and purpose of the study and unwillingness to co-operate with the Investigator; 4) patients who have used any drug affecting GI motility or intestinal microbiota during the previous 4 weeks.

According to previous pediatric studies about functional gastrointestinal disorders that included subjects with at least 8 years of age, we divided the study population into <8 and >8 years [16,17].

**Figure 1:**
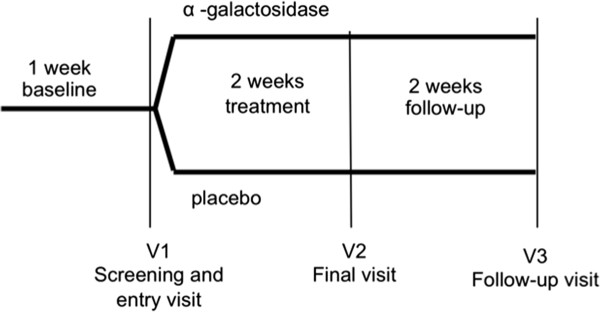
Study flow chart.

Each potentially eligible patient was invited to discuss the study in detail. Figure 1 shows the patient flow.

After informed consent, eligible patients entered a baseline period of 1 week during which they recorded data on a daily basis in a questionnaire/diary provided at study entry by the physician. At the end of the baseline period, patients returned to the centre where they were randomized in a double-blinded fashion to receive placebo (n = 25) or α-galactosidase (n = 27) (Sinaire, Promefarm) according to a computer-generated random allocation. The dose was provided by the manufacturer in the investigator brochure and is proportional to the adult dose based on body weight class. Both treatments were given as drops or tablets according to body weight: 4 or 8 drops were administered to children with body weight lower than 20 kg and to children with body weight between 20 and 40 kg, respectively. One tablet was administered to children with body weight over 40 kg. The treatment was administered at the beginning of each meal 3 times a day for 2 weeks. No medications were allowed for the duration of the study. As part of the initial evaluation, a careful dietary history, including assessment of fibre intake, was conducted. No changes in any of the dietary habits of the patients were allowed throughout the duration of the study. Children underwent follow-up 2 weeks after the end of treatment. We have evaluated the global distress associated with gas-related symptoms (bloating, flatulence, abdominal distension and abdominal spasms) by means of validated visual score, i.e., Faces Pain Scale-Revised (FPS-R). The FPS-R has been shown to be reliable for assessment of the intensity of children’s pain age 4 or 5 onward [18]. A parent-report assessment was performed for children 4 years of age and older (but less than 10 years), while a self-report assessment was performed for children and adolescents 10 years of age and older. Parents and/or patients recorded symptoms 3 times daily (7–8 am, 2–3 pm, 6–7 pm) during the treatment. It is well established that relatively few symptoms may be recorded by diaries and ratings can be performed only at a fixed time [19]. Bloating, flatulence (wind), visible distension and spasms were rated (0 = absent; 5 = very severe) according to intensity and frequency of episodes by parent and/or child using a daily diary chart. Scores of 4 or more were associated to severe bloating. Symptomatic episodes with a score of 2 or more defined a significant flatulence.

A “physician’s overall evaluation” was made by simple questions addressed to the child/parent “How did you feel during the treatment as compare to the period before?” at the end of treatment and “How did you feel as compared to the treatment period?” at the end of follow-up and asked to select a category response as follows: completed or marked improvement, mild improvement, unchanged or worse.

The primary outcome measure was reduction in the FPS-R at the end of treatment compared to baseline. The reduction in proportion of patients with relevant symptoms is the secondary outcome measure.

The study protocol was defined in accordance with the Declaration of Helsinki and approved by the ethical committee of the University Hospital Umberto I in Rome. Written informed consent was obtained from parents of all children; children over 12 years of age signed a statement of assent.

### Statistical analysis

By estimating for the primary endpoint a standard deviation of 1.25 and a targeted difference in the mean scores between groups of 1, a type-I error of 5%, a power of 80% and a drop-out rate of 5%, the number of patients to be enrolled in each group was 26.

All characteristics were summarized by usual descriptive statistics such as mean and standard deviation for continuous variables and rates for categorical variables. Two-sided t-test was used to compare the means of continuous variables; chi square test was used to compare the rates of categorical measures.

Values of p-value smaller than 0.05 were considered statistically significant.

## Results

**Figure 2:**
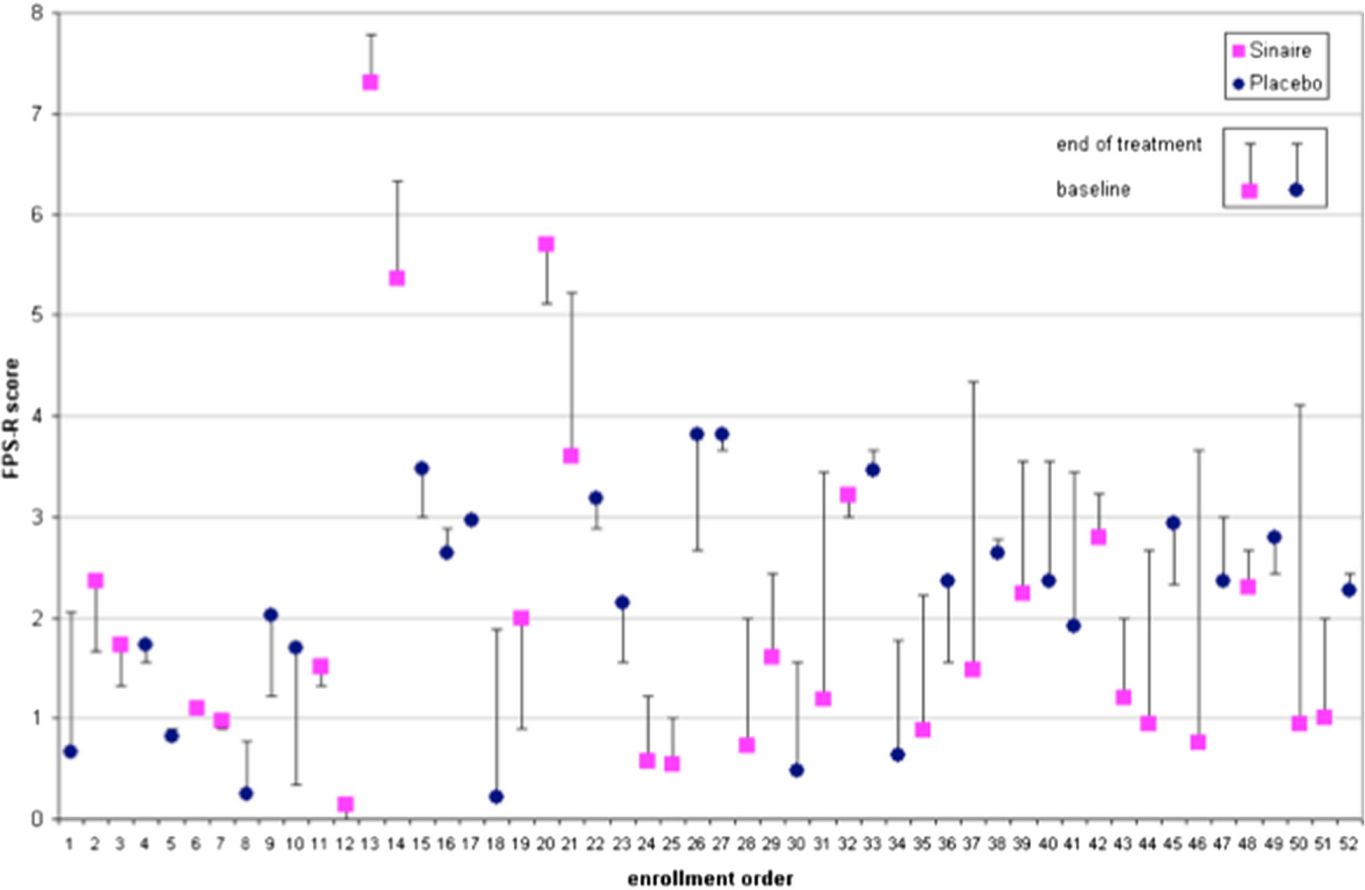
Change of FPS-score in any patient before and after the treatment.

Fifty-two patients completed the trial (32 females, median age 8 yrs, range 4–17 yrs). The demographic and baseline data did not differ between the two study groups (Tables 1 and 2). The mean FPS-R decreased more in the α-galactosidase than placebo group; the difference between the FPS-R score change in the two groups was statistically significant (p = 0.02) (Table 2). The FPS-R score improved more in the α-galactosidase than placebo group, even if the difference was not statistically significant (Table 3). Only in 3 (11%) patients treated with α-galactosidase, FPS-R score worsened by more than 0.5 units (Figure 2).

**Table 1:** Demographic features

**Variable**	**α-galactosidase**		**placebo**		**P**
	**(N = 27)**		**(N = 25)**		
**Demographic data**					
Gender: n (%)					
male	11	(41%)	9	(36%)	0.73
female	16	(59%)	16	(64%)	
Age (yrs): n (%)					
<8	12	(44%)	10	(40%)	0.75
> = 8	15	(56%)	15	(60%)	
Weight (kg): n (%)					
≤20	9	(33%)	7	(28%)	0.92
>20, ≤40	7	(26%)	7	(28%)	
>40	11	(41%)	11	(44%)	

**Table 2:** Rates of symptoms and Global Distress assessed by FPS-R at baseline and after treatment

**Symptoms**	**Baseline**			**At the end of treatment**		
	**α-galactosidase**	**Placebo**	***p*-value**	**α-galactosidase**	**Placebo**	***p*-value**
	**(N = 27)**	**(N = 25)**		**(N = 27)**	**(N = 25)**	
Flatulence*: n, (%)	16 (59%)	12 (48%)	*p* = 0.42	5 (19%)	12 (48%)	p = 0.02^
Abdominal distension**: n, (%)	12 (44%)	14 (56%)	*p* = 0.41	8 (30%)	11 (44%)	p = 0.36
Abdominal spasm***: n, (%)	14 (52%)	14 (56%)	*p* = 0.76	4 (15%)	4 (16%)	p = 0.91
FPS-R^†^, mean ± SD	2.8 ± 1.8	2.3 ± 0.9	*p* = 0.22	2.0 ± 1.7	2.1 ± 1.1	p = 0.73
Difference between FPS-R at baseline and after treatment				−0.8 ± 1.1	−0.2 ± 0.8	p = 0.02^
Number of days with severe bloating during treatment (mean ± SD)				3.4 ± 3.6	5.4 ± 3.4	p = 0.03^

*patients with significant episodes (score of > 2) of flatulence ≥ 3 days/week.

**patients with visible abnormal distension ≥ 3 days/week.

***patients with abdominal spasm ≥ 3 days/week.

^†^mean of 21 measurements for each patient.

^p value < 0.05 was considered statistically significant.

**Table 3:** Rates of patients in whom FPS-R improved or worsened by more than 0.5 units

**Variable**	**α-galactosidase**		**placebo**		**P**
	**(N = 27)**		**(N = 25)**		
Pts with difference of FPS-R score < −0.5, n (%)	14	(52%)	8	(32%)	0.15
Pts with difference of FPS-R score > 0.5, n (%)	3	(11%)	6	(24%)	0.22

The number of days with moderately to severe bloating was significantly lower in the α-galactosidase than placebo group (p = 0.03) (Table 2).

The trend for worse score along the day was common for both groups. We did not find significant differences between the active and placebo at any single time point: bloating scores were better in the morning (α-galactosidase 1.8 ± 1.5, placebo 2.1 ± 1.2), worse in the afternoon (α-galactosidase 2.1 ± 1.7, placebo 2.1 ± 1.1) and in the evening (α-galactosidase 2.1 ± 1.9 placebo 2.3 ± 1.1). The scores were lower with α-galactosidase even if no difference was statistically significant.

α-galactosidase reduced the proportion of patients with significant flatulence compared to placebo during treatment (p = 0.02) but no difference was seen for abdominal distension and spasms (Table 2). Similarly, no change was observed in the daily bowel movements after treatment with α-galactosidase (0.9 ± 0.4) and placebo (1.0 ± 0.3) as compared to baseline (α-galactosidase 1.0 ± 0.4; placebo 1.1 ± 0.5).

**Figure 3:**
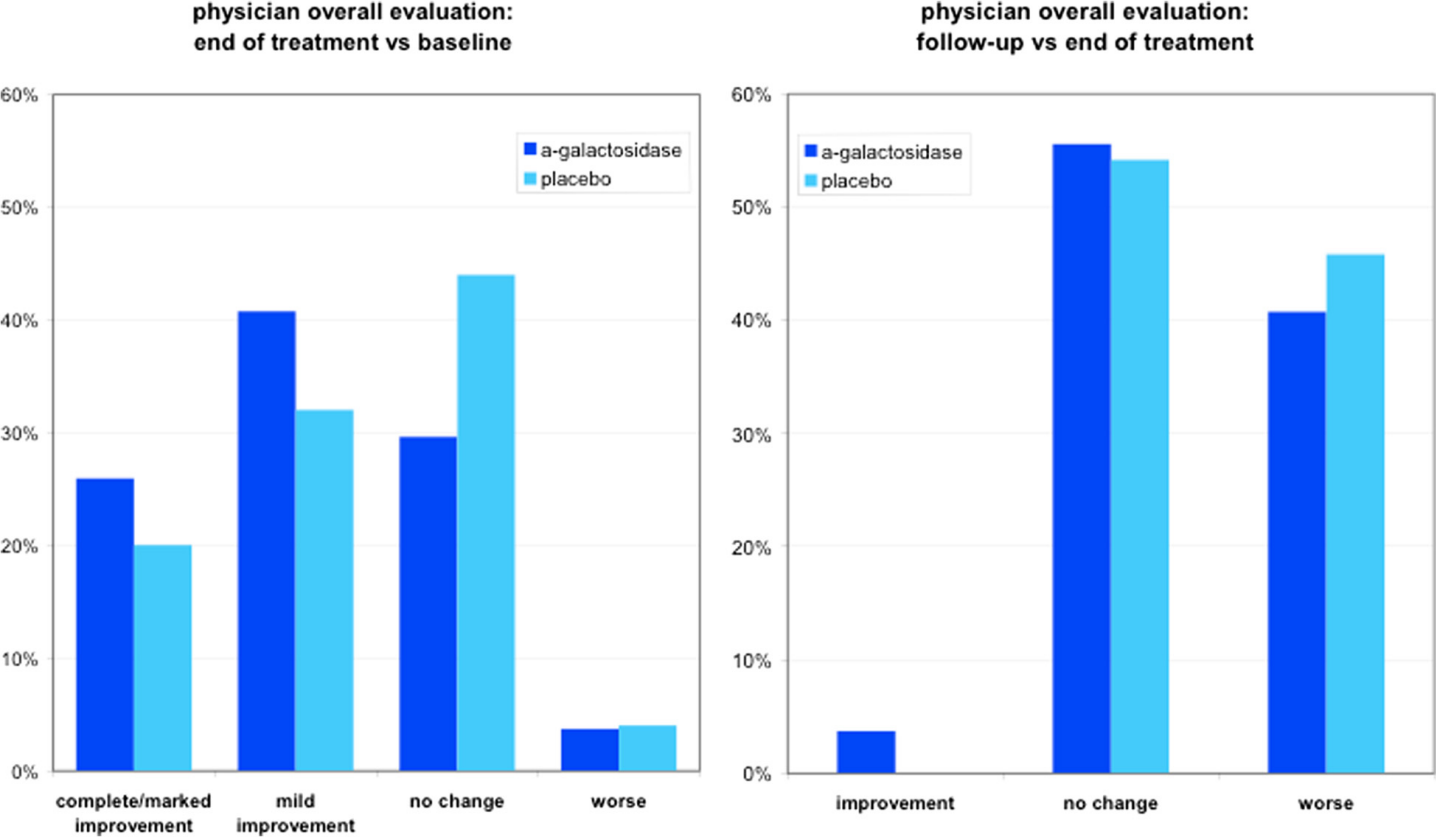
Physician overall evaluation at end of treatment and at follow-up.

According to physician’s overall evaluation, children receiving α-galactosidase had higher rates of improvement (67%) than placebo (52%) but the difference was not statistically significant. At the end of follow-up, about half the patients in both groups did not change and about 40% of patients in both groups worsened with no significant difference in both groups (Figure 3).

Both treatments were well tolerated. No adverse effects were reported during the study.

## Discussion

This randomized placebo-controlled study provides evidence that oral α-galactosidase can improve gas-related symptoms in children and adolescents. The efficacy of α-galactosidase was supported by a significant and parallel decrease in the severity of bloating and FPS-R score. We used the FPS-R scale in order to measure the overall degree of discomfort associated with gas-related symptoms in everyday life. This could be considered acceptable as, in our patients, bloating was the main symptom whilst abdominal pain/spasm and altered bowel habit were only occasionally present.

The significant decrease of FPS-R score occurring in the active group and the marginal change in the placebo group provides the evidence of the efficacy of α-galactosidase. The similar score values at end of treatment should be evaluated taking into account the apparent imbalance in the baseline FPS-R.

The significant effect on bloating was also confirmed by a reduction in the number of days with severe episodes of bloating. In the opinion of the authors, 2 less days of bloating over the week in the alpha-galactosidase group as compared to placebo would be clinically significant.

Gas-related symptoms are common medical problems in pediatrics; the subjective sensation of bloating with or without a visible increase of abdominal distension is one of the most common complaints faced by pediatric gastroenterologists and general pediatricians in their clinical practice. Gas-related symptoms are included in the functional gastrointestinal disorders (FGID) as there is no evidence of morphological or biochemical abnormalities. When the symptoms are mild or episodic, reassurance and dietary changes may be sufficient. However when children suffer of recurring symptoms of bloating, flatulence (wind), distension and abdominal discomfort/pain with significant impairment for children and their family, it is reasonable to have a more proactive approach to reduce symptoms. The proposed pathophysiology of gas-related symptoms includes a variety of different and overlapping mechanisms, which are difficult to recognize in clinical practice [4,5].

Bloating and distension together with belching, aerophagia and flatulence, have been attributed to excessive intestinal gas accumulation, impaired handling of gas in the small intestine, impaired clearance from the proximal colon, psychological factors, altered gut microflora, incomplete digestion and malabsorption of carbohydrates [20].

We did not investigate the dietary habits of children before and during our study. Parents were instructed to continue with their usual diet over the study period in order to avoid any possible impact from a change of diet to the study results. According to clinical history and parents’ opinion, the symptoms were apparently related to meals: a fact which is also demonstrated by the bloating severity score which was lowest at the time of waking up and steadily increased during the day.

There are different strategies that may reduce intestinal gas including active charcoal, simethicone etc. [9,10]. Recently, the use of antibiotics seems to provide significant relief of functional GI symptoms, bloating and abdominal pain, but the need for repeated treatment cycles may have an important impact on the intestinal bacterial flora [21,22], especially in children.

In recent years, an alternative approach, based on the administration of *α*-galactosidase, an enzyme with amylase-like activity, seems to be effective in reducing the production of intestinal gas by breaking down non digestible oligosaccharides (NDO) before they reach the lower intestine. Indeed, the human intestine does not contain the enzyme required to digest NDO and their consequent incomplete digestion produces increased bacterial fermentation generating hydrogen, methane and carbon dioxide [13-15].

Our results show that in children with predominant gas-related symptoms, the administration of *α*-galactosidase significantly reduces global distress, bloating and flatulence compared to placebo, but does not significantly reduce other symptoms such as abdominal distension and spasms which generally occur in functional gastrointestinal disorder. However this is consistent with the mechanism of action of *α*-galactosidase. The reduced formation of gas by colonic bacteria may have been expected to have other beneficial effects on distension of colon segments and abdomen.

The strength of our findings is mitigated by the findings in some secondary efficacy endpoints. No significant changes were observed between *α*-galactosidase and placebo for the “physician’s overall evaluation” at end of treatment and at follow-up. This may be related to the fact that this global evaluation may include other symptoms (in addition to bloating) which are poorly responsive to *α*-galactosidase such as symptoms that are not gas-related. The effect of *α*-galactosidase on gas related symptoms does not appear to be long-lasting. The improvement observed at the end of treatment was not sustained after 2 weeks of follow-up but this is consistent with the mechanism of action of the enzyme.

The limitations of our study are the short duration of treatment (2 weeks only) and the relatively small sample compared to trials in adult populations.

There is not much information about placebo response on gas related symptoms in children in literature. In our study, we did not see apparent difference in the placebo response between children of less and above 8 years. There were no reported adverse effects during the study. Based on our study there is no safety issue related to the use of *α*-galactosidase in children with bloating and gas-related symptoms. This is also supported by clinical practice and post-marketing experience on the use of *α*-galactosidase in children as well as in adults.

Oral *α*-galactosidase was effective in the short-term treatment of gas-related symptoms in children who were referred to specialist care. Its use is also supported by non-toxicity, good tolerability and availability of the formulation in drops, suitable for pediatric patients.

Further longer and larger randomized controlled clinical trials are needed to assess the efficacy and usefulness of *α*-galactosidase in children with gas-related symptoms and to identify subgroups of patients who are more likely to respond (or fail) to this agent.

A detailed medical history may help physicians to recognize children in whom, based on the medical history, food appears to induce or favor gas-related symptoms.

A symptomatic treatment approach for these children could be beneficial: if there is no significant improvement in the main target symptom or overall symptoms after 2 weeks, a different alternative approach should be considered.

This study was placebo-controlled, as currently there is no reference treatment for gas-related symptoms in FGID. The evidence of efficacy of antiflatulents such as simethicone and activated charcoal is weak, together with that of probiotics, such as different strains of Lactobacillus or Bifidobacterium spp. Similarly, there does not appear to be any robust evidence for using antispasmodics, prokinetics or non-absorbable antibiotics if the target symptom is bloating. Our study was carried out in children who predominantly had bloating and gas-related symptoms, with no significant abdominal pain. Consequently, the beneficial results obtained in this study cannot be considered valid for children suffering from different variants of functional gastrointestinal disorder (IBS-predominant constipation or IBS-predominant diarrhea).

We acknowledge the limitations of the present study. The number of included patients is limited and their age range is wide. In addition, although the “gas-related syndrome” may be considered as a functional digestive syndrome characterized by non specific gastrointestinal symptoms, including bloating, flatulence, abdominal distension and discomfort that the patient attributes to an excess of abdominal gas, however this condition is poorly defined and overlaps with IBS. All our patients meet Rome III criteria for IBS and the American College of Gastroenterology IBS Task Force recommends that further investigations are unnecessary in young patients without alarm features with the exception of celiac disease serology. However, different diagnoses, including lactose intolerance, fructose intolerance, small bowel bacterial overgrowth and aerophagia, were not formally investigated in our patients. Finally, although we instructed the parents of our patients to continue with their usual diet over the study period, the dietary habits of our children were not formally recorded in diary during the study. For this reason, we can not relate the symptoms to the type of diet (high and low fiber intake).

This study has shown for the first time that pediatric patients with predominant gas-related symptoms had a better response than placebo in the short term use of oral *α*-galactosidase. The improvement of symptoms becomes evident in a few days, in particular the reduction of the severity and frequency of bloating and flatulence. This effect tends to disappear in half of patients 2 weeks after treatment withdrawal.

## Conclusions

In conclusion, oral *α*-galactosidase was effective and very well tolerated in the treatment of bloating and gas-related symptoms in children and adolescents aged 4 – 17 years.

## Competing interests

The authors declare that they have no competing interests.

## Authors’ contributions

DNG and OS designed the study and wrote the manuscript. MS and FF followed-up patients. CC, BG and AM designed the study. CS is the head of the Pediatric Gastroenterology Unit, approved the study design and strongly revised a draft of the paper. Guarantor of the article: CS. All authors read and approved the final manuscript.

## Pre-publication history

The pre-publication history for this paper can be accessed here:

http://www.biomedcentral.com/1471-230X/13/142/prepub
